# Calcification-driven CO_2_ emissions exceed “Blue Carbon” sequestration in a carbonate seagrass meadow

**DOI:** 10.1126/sciadv.abj1372

**Published:** 2021-12-15

**Authors:** Bryce R. Van Dam, Mary A. Zeller, Christian Lopes, Ashley R. Smyth, Michael E. Böttcher, Christopher L. Osburn, Tristan Zimmerman, Daniel Pröfrock, James W. Fourqurean, Helmuth Thomas

**Affiliations:** 1 Institute of Carbon Cycles, Helmholtz-Zentrum Hereon, Geesthacht, Germany.; 2 Geochemistry and Isotope BioGeoChemistry Group, Department of Marine Geology, Leibniz Institute for Baltic Sea Research, Warnemünde, Germany.; 3 Institute of Environment, Department of Biological Sciences, Florida International University, Miami, FL, USA.; 4 Soil and Water Sciences Department, Tropical Research and Education Center, University of Florida, Homestead, FL, USA.; 5 Marine Geochemistry, University of Greifswald, Friedrich-Ludwig-Jahn Str. 17a, D-17489 Greifswald, Germany.; 6 Department of Maritime Systems, Interdisciplinary Faculty (INF), University of Rostock, Albert-Einstein-Straße 21, D-18059 Rostock, Germany.; 7 Department of Marine, Earth and Atmospheric Sciences, North Carolina State University, Raleigh, NC, USA.

## Abstract

Long-term “Blue Carbon” burial in seagrass meadows is complicated by other carbon and alkalinity exchanges that shape net carbon sequestration. We measured a suite of such processes, including denitrification, sulfur, and inorganic carbon cycling, and assessed their impact on air-water CO_2_ exchange in a typical seagrass meadow underlain by carbonate sediments. Eddy covariance measurements reveal a consistent source of CO_2_ to the atmosphere at an average rate of 610 ± 990 μmol m^−2^ hour^−1^ during our study and 700 ± 660 μmol m^−2^ hour^−1^ (6.1 mol m^−2^ year^−1^) over an annual cycle. Net alkalinity consumption by ecosystem calcification explains >95% of the observed CO_2_ emissions, far exceeding organic carbon burial and anaerobic alkalinity generation. We argue that the net carbon sequestration potential of seagrass meadows may be overestimated if calcification-induced CO_2_ emissions are not accounted for, especially in regions where calcification rates exceed net primary production and burial.

## Teaser

Rigorous carbon accounting shows that calcification-driven CO_2_ emissions can exceed seagrass “Blue Carbon” storage.

## INTRODUCTION

Seagrass ecosystems are some of the most organic carbon (OC)–dense systems on Earth, and it has been argued that OC sequestration here is disproportionately large in comparison to other terrestrial and marine ecosystems, thus constituting an important sink in the global carbon cycle (*1*, *2*). Presently, there exists an apparent consensus that the protection and enhancement of such “Blue Carbon” storage in seagrass meadows is an effective strategy to mitigate increasing atmospheric CO_2_ levels (*3*–*7*). However, biogeochemical cycling in seagrass ecosystems is complex, and many other processes exist, which may counteract net OC sequestration. These processes collectively regulate local budgets of dissolved inorganic carbon (DIC) and total alkalinity (TA), and include ecosystem calcification (*8*–*10*) and anaerobic metabolism (*11*). When net TA production occurs, the resulting carbonate system reequilibration consumes CO_2_, which is then compensated by net CO_2_ uptake from the atmosphere. The impact of these TA-generating processes [namely, iron (Fe), sulfate (SO_4_^2−^), and nitrate (NO_3_^−^) reduction] on net CO_2_ uptake depends on the ultimate fate of the metabolic products produced. Net TA generation will only occur if these metabolic products are permanently removed in their reduced form [via long-term burial of reduced Fe or sulfur (S) or release of N_2_ gas following denitrification] but will not if the reduced species are reoxidized in place. Competing with these TA sources, a key sink for TA (source of CO_2_) in Blue Carbon habitats is the precipitation of carbonate minerals (*5*, *12*, *13*). This is especially so in tropical and subtropical seagrasses, which play a disproportionately large role in the global carbonate cycle (*14*). The impact of these internal reworkings of S, Fe, N, and carbonate minerals on surface water TA/DIC and, ultimately, air-sea CO_2_ exchange remains largely unknown. This has been identified as a key gap in our understanding of the role of Blue Carbon ecosystems in the global carbon cycle (*10*, *15*).

Despite the understanding that carbonate precipitation generates CO_2_, carbonate seagrass meadows are still considered as important Blue Carbon sinks based largely on the assumption that these carbonate minerals were formed elsewhere (*10*, *12*), or that the CO_2_ produced by calcification is rapidly consumed by photosynthesis (*16*). As a result, policies aimed at restoring or protecting Blue Carbon habitats have gathered momentum as an approach to fight climate change while also achieving co-benefits of habitat improvement (*4*, *5*, *17*). In recent Intergovernmental Panel on Climate Change (IPCC) reports, these ocean-based mitigation tools have been assigned a high chance of success (IPCC Chapter 5.5.1.2.2) (*18*), despite the lack of empirical evidence demonstrating net CO_2_ uptake by carbonate seagrass meadows (*13*). Still, uncertainty in the scientific community regarding the role of calcification in Blue Carbon mitigation is explicitly acknowledged by the IPCC, and its timely resolution is seen as “highly desirable” (*18*). Blue Carbon mitigation requires public and private buy-in (*4*, *19*), and a general understanding that the actions taken will indeed enhance net carbon sequestration. Many of these Blue Carbon habitats are managed by resource-limited nations, introducing a climate injustice risk, as there exists a perception that they are being asked to carry the climate burden of more industrialized nations (*20*). It is therefore crucial that these ocean-based management actions only be enacted for Blue Carbon habitats where increased carbon sequestration is plausible.

To address these uncertainties, we used atmospheric eddy covariance (EC) to directly measure air-water CO_2_ exchange in Florida Bay, USA, one of the largest seagrass-dominated estuaries in the world, and a known OC sink (*21*). We combine these EC fluxes with geochemical approaches to attribute the major processes contributing to source or sink behavior. We sampled at three locations in close proximity within this seagrass meadow, representing regions of (i) high seagrass aboveground biomass [high density (HD)], (ii) low seagrass density (LD), and (iii) bare sediment (B). The selection of these sites was intended to characterize the seagrass meadow occupying the EC footprint. We combined pore-water and solid-phase analyses with a continuous-flow incubation that either included or excluded living seagrass biomass. This approach lets us ascribe changes in surface water TA and DIC to net process rates in the sediment, which were, in turn, integrated into a biogeochemical budget, assessing their effects on air-water CO_2_ uptake or release.

## RESULTS

### Net CO_2_ emissions associated with carbonate mineral dissolution/reprecipitation

**Fig. 1.**
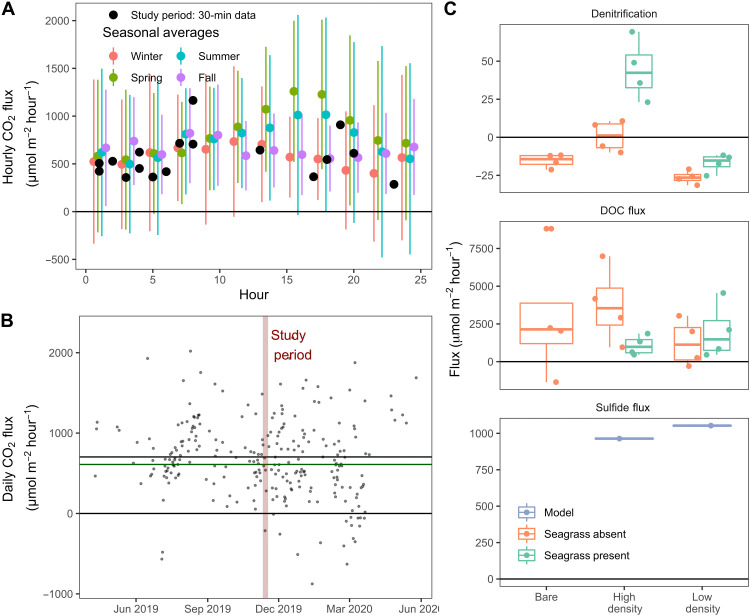
Summary of measured air-water and sediment-water fluxes. (**A**) Diel trend in CO_2_ flux presented as discrete 30-min measurements during the study period (black circles) and annual mean fluxes for the year surrounding the study period, binned in 2-hour intervals [colored circles (x ± SD)]. (**B**) Daily mean CO_2_ fluxes for the year surrounding the study period, where the time frame of the 1-week study period is highlighted in red, and mean CO_2_ fluxes from the study period and annually are shown as the green and black horizontal lines, respectively. (**C**) Average net N_2_ flux (denitrification), dissolved OC (DOC) fluxes, and sulfide fluxes, separated by site (bare, HD, and LD) along the *x* axis. Note that all sulfide measurements from the continuous-flow experiment were below the limit of quantification, so only fluxes calculated in PROFILE are presented here. By convention, release from sediments or water is reported as positive fluxes. All error bars represent x ± SD.

Direct EC measurements reveal this seagrass meadow as a moderate source of CO_2_ to the atmosphere (Fig. 1, A and B), with average emissions during the ~1-week study period (10 to 18 November 2019; red vertical line in Fig. 1B) of 610 ± 990 μmol m^−2^ hour^−1^ (green line in Fig. 1B; mean ± 1 SD of 30-min records). This is slightly below the previously reported summer-time CO_2_ flux for this site (972 ± 612 μmol m^−2^ hour^−1^) (*22*) and just below the annual average (700 ± 660 μmol m^−2^ hour^−1^ or 6.1 mol m^−2^ year^−1^; black line in Fig. 1B). While annual hourly climatology demonstrates a clear diel trend in the warmer spring and summer months, with greater CO_2_ emissions during the afternoon [Fig. 1A, green (spring) and blue (summer) circles], such a day-night difference was not present during the study period, which occurred in the fall (Wilcoxon, *P* = 0.61; Fig. 1A, black circles). These seasonal trends are consistent with the hypothesis that factors other than seagrass net ecosystem metabolism control the CO_2_ system at this site, contrasting with previous studies describing the importance of water column productivity for seasonal CO_2_ uptake in fall (*23*, *24*). In the case of air-water CO_2_ flux, this nonmetabolic forcing includes the role of temperature in enhancing convection-mediated air-water gas transfer (*22*). These CO_2_ emissions are likely an underestimate of the global warming potential of this seagrass meadow, which is likely enhanced by the release of other greenhouse gases (*25*) like CH_4_ and N_2_O.

**Fig. 2.**
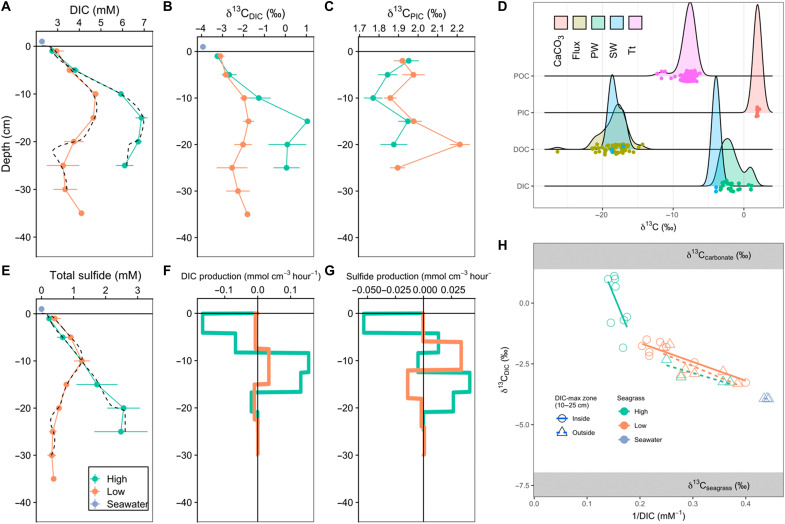
Summary of sediment geochemical and isotopic results. (**A** to **C**) Vertical profiles of pore-water DIC, δ^13^C_DIC_, and δ^13^C_PIC_. (**D**) Carbon isotopic signatures for selected pools, including inorganic carbon (CaCO_3_), pore-water (PW), surface water (SW), *Thalassia testudinum* (Tt), and DOC from the flow-through flux experiment (Flux). Individual data points and a smoothed density plot (ggridges version 0.5.3) help to visualize the distribution for each isotopic endmember. Total dissolved sulfide (**E**) and modeled production rates for DIC and total sulfide (**F** and **G**). (**H**) “Keeling plot” of pore-water δ^13^C_DIC_. Model output pore-water concentrations are shown in black dotted lines in (A) and (B) (each core was modeled individually).

**Fig. 3.**
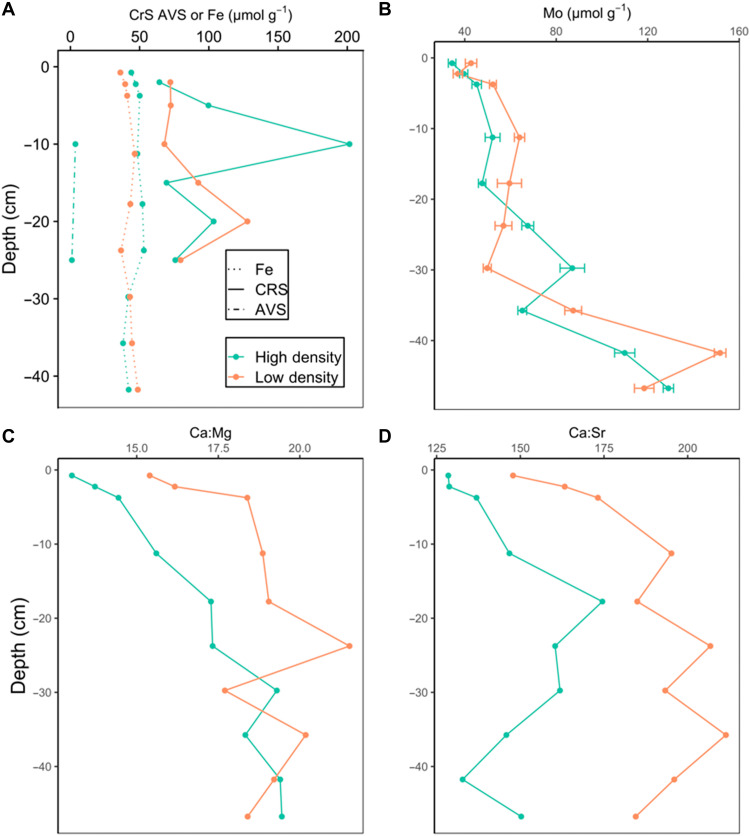
Vertical profiles of solid-phase geochemical data. Reported here are total Fe, chromium-reducible sulfur (CRS), and acid-volatile sulfide (AVS) (**A**); total Mo (**B**); and molar ratios of Ca:Mg and Ca:Sr (**C** and **D**).

In line with strong carbonate dissolution, we observed notable excesses of pore-water DIC coinciding with a moderate (LD) or large (HD) enrichment of ^13^C in DIC such that average pore-water δ^13^C_DIC_ (−1.7 ± 1.4‰) was at least 2‰ “heavier” than surface water (δ^13^C_DIC_ = −3.9 ± 0.02‰) (Fig. 2). For sediment depths outside of the zone of peak DIC accumulation, a Keeling plot points toward an isotopic endmember of ~0‰, characteristic of “heavy” DIC from carbonate mineral dissolution mixed with “light” respiratory DIC from dissolved OC (DOC) degradation (δ^13^C_DOC_ = −18 ± 1.9‰). However, within the DIC maximum zone in the HD cores, DIC was enriched in ^13^C, with an average δ^13^C_DIC_ of 0.0 ± 1.1‰ (−2.1 ± 0.6‰ in the LD cores). The isotopic endmember indicated for this “DIC-maximum zone” is well above measured particulate IC (PIC) δ^13^C_PIC_ of 1.9 ± 0.12‰. Enrichment in ^13^C of this magnitude cannot be explained solely by CaCO_3_ dissolution. Rather, this enrichment necessitates the consumption of isotopically light pore-water DIC by carbonate precipitation under quasi–closed-system conditions (*26*). This hypothesis is supported by the modeled DIC production rates (Fig. 2F), where net DIC generated below 10 cm depth is consumed in the upper 10 cm of sediment by combined autotrophic sulfide oxidation and carbonate reprecipitation. Likewise, decreased solid-phase Ca:Sr and Ca:Mg ratios in the upper 10 to 20 cm of sediment support carbonate mineral recrystallization in this region (Fig. 3, C and D). Our findings are consistent with previous work showing this coupled dissolution/reprecipitation to be highest when seagrasses are especially dense (*26*, *27*). This internal carbonate recycling is critically important to the overall C cycle, as it regulates the burial efficiency of CaCO_3_, ultimately governing CO_2_ emissions from the sediment related to net carbonate burial.

### Denitrification and sulfate reduction are only minor alkalinity sources

Pore-water total dissolved sulfide was elevated relative to surface water (Fig. 2E), especially in the HD cores, and was generated by net sulfate reduction at depths of ~10 cm (LD) to ~15 cm (HD) (Fig. 2G). This excess sulfide corresponded to a large modeled vertical sulfide flux (Fig. 1C) that was presumably reoxidized in the narrow oxic zone of the sediments or the water column. All dissolved sulfide samples from the sediment flux incubation were below the limit of detection, confirming that reduced sulfur diffusing upward is quantitatively lost to either (i) oxidation to sulfate or (ii) burial as FeS_2_ + S^0^ (Fig. 3A), or (iii) burial as FeS. We expect that sulfide oxidation is largely limited to depths of 0 to 30 cm, as the relative increase in Mo (a redox-sensitive tracer) below 30 cm depth (Fig. 3B) indicate permanent anoxia below this threshold. While bulk sediments of the upper 0 to 30 cm are also anoxic, the relative Mo depletion in this zone supports localized and sporadic oxidation in the millimeters surrounding seagrass roots. The impact of net sulfate reduction on TA depends on the fate and form of net reduced sulfur burial. To this point, we observe substantial accumulation of chromium (II)–reducible sulfur (CRS; FeS_2_ + S^0^), with an average content of 94 ± 37 μmol CRS g^−1^ (0.3 ± 0.12% dry weight), and a peak of 200 μmol g^−1^ in the peak sulfate reduction zone of site HD. Lower concentrations of acid-volatile sulfide (AVS) (maximum of 3.6, average of 2.4 ± 1.8 μmol g^−1^) indicate a relatively minor contribution of H_2_S and FeS to total sulfur burial. Because the total Fe content (44.9 ± 5.3 μmol g^−1^) is half of average CRS, at most ~100% of reduced sulfur may be buried as pyrite (FeS_2_). However, previous work (*28*) shows that 50% or more of the sedimentary Fe pool in central Florida Bay is not directly associated with CRS, suggesting substantial burial of elemental sulfur (S^0^) as a product of partial sulfide reoxidation (*29*). This is supported by the CRS peak at 10 cm at the HD site, possibly caused by sulfide oxidation to sulfur at the surface of seagrass roots (*30*). Assuming that CRS is composed of equal proportions FeS_2_ and S^0^, net sulfate reduction and burial will produce TA and DIC in a ratio of 2:1 (Table 2).

Continuous-flow incubations conducted in the dark showed that denitrification was closely balanced by N_2_ fixation (Fig. 1C), and only cores from the HD site with seagrass biomass included were, on average, net denitrifying (positive N_2_ flux). In contrast, all other cores were net N_2_ fixing (negative N_2_ flux), despite dark conditions favorable to net denitrification (Fig. 1C) (*31*). Averaged across all cores, net denitrification was not significantly different from zero (x = −3 ± 27 μmol m^−2^ hour^−1^), consistent with the understanding that denitrification in seagrass meadows is spatially heterogeneous, tends to covary with autochthonous organic matter input (*32*, *33*), and is part of a highly conservative N cycle. Our low denitrification measurements are also consistent with the low nitrate concentrations typical of many carbonate seagrasses (*34*), where all available nitrate appears to be quantitatively denitrified. That net denitrification was only observed in cores from the HD site containing living seagrass biomass (Fig. 1C), where pore-water sulfide was also maximized (Fig. 2E), contrasts with the expected sulfide inhibition of denitrification in Florida Bay (*33*). Instead, denitrification may have been enhanced by the leaching of labile OM (organic matter) leaching from the seagrass rhizosphere. Likewise, the dissolution of carbonate sediments, as expected during this dark/heterotrophic incubation, is a potential abiotic source of dissolved OM through the release of carbonate-associated organic matter (*35*). We posit that these abiotic and biotic OM sources together drove our large measured DOC fluxes. Broadly, the observation of strong internal recycling with respect to N and S supports the notion that alkalinity dynamics in this carbonate seagrass meadow are dominated by CaCO_3_ precipitation and dissolution, rather than anaerobic alkalinity generation via nitrate, sulfate, or metal oxi(hydroxi)de reduction.

### CO_2_ emissions are driven by net CaCO_3_ production

**Fig. 4.**
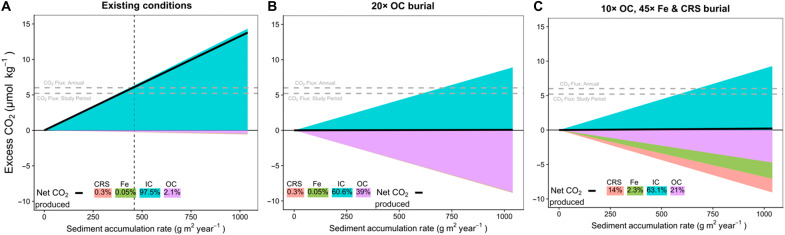
Modeled excess CO_2_, across a range in literature sediment accumulation rates. In (**A**), sediment fractions of CRS, Fe, IC, and OC were set according to direct measurements from this study. In (**B**), the OC fraction was artificially increased until excess CO_2_ remained at 0 across the range in sediment accumulation such that CO_2_ released by calcification was offset by CO_2_ consumed by OC burial and Fe and SO_4_ reduction. In (**C**), net CO_2_ balance was achieved by increasing OC, Fe, and CRS fractions by factors of 10, 45, and 45, respectively.

We detected consistent net CO_2_ emissions from this seagrass meadow of 610 μmol m^−2^ hour^−1^ over the study period and 700 μmol m^−2^ hour^−1^ (6.1 mol m^−2^ year^−1^) as an annual average (Fig. 1B). This is notable given the consensus impression of seagrasses as net Blue Carbon sinks, and in light of our understanding of these seagrasses as being net autotrophic (*36*). That CO_2_ emissions persisted at this site, despite seagrass ecosystem productivity and known net OC burial (*21*), suggests that processes besides seagrass net metabolism drive air-water CO_2_ exchanges. To find the dominant carbon source for these CO_2_ emissions, we constructed a simple TA and DIC budget, considering the impact of calcification (IC accumulation), net Fe^3+^ and SO_4_^2−^ reduction (CRS), and net OC burial. Measured denitrification was not significantly different from 0; hence, it was excluded as a putative TA source. CO_2_ consumption by net OC accumulation reduced excess CO_2_ by 3.4%, while the burial of CRS and Fe (i.e., net SO_4_^2−^ and Fe^3+^ reduction) only reduced excess CO_2_ by ~1%. When these TA/DIC consuming and producing processes are summed, we find that excess CO_2_ available for release to the atmosphere (solid black line in Fig. 4A) increases with sediment accumulation. This excess CO_2_ generated by IC accumulation explains the remaining 95.8% of the change in excess CO_2_ (Fig. 4A). Using an average gas transfer velocity of 11.7 cm hour^−1^, our measured CO_2_ fluxes can be converted to excess CO_2_ values of between 6.0 and 5.2 μmol kg^−1^ (gray dashed lines in Fig. 4, A to C), well within the range of excess CO_2_ in the budget. Furthermore, the sediment accumulation rate (SAR) required to sustain measured annual average CO_2_ emissions (700 ± 660 μmol m^−2^ hour^−1^ or 6.1 mol m^−2^ year^−1^) is only 460 g sediment m^−2^ year^−1^ (vertical dotted line in Fig. 4A), intermediate of literature values for central Florida Bay (*37*, *38*).

To find the rate of OC burial required to balance the CO_2_ produced by calcification, we repeatedly ran the model, increasing the fraction of sediment composed of OC until total excess CO_2_ was equal to 0. For these processes to balance out, the sediment OC fraction would need to increase by a factor of nearly 20, from 2.1 to 39% (compensated by IC decrease from 97.5 to 60.6%; Fig. 4B). OC contents of this order are much greater than those that can be found in carbonate seagrass meadows elsewhere in Florida Bay (*39*) or globally (*40*, *41*). In a final model, we increased OC burial by only one order of magnitude (to 21%), but increased CRS and Fe fractions by a factor of 45, simulating an artificial Fe amendment scenario. In this admittedly unrealistic scenario, CO_2_ emissions due to calcification are balanced approximately equally by OC burial and anaerobic TA generation by net sulfate and Fe reduction. In these additional scenarios (Fig. 4, B and C), some unknown additional CO_2_ source would be required to bring modeled excess CO_2_ (thick black line) in line with the excess CO_2_ associated with measured CO_2_ emissions (gray dashed lines). As nearly one-third of seagrass meadows (*12*) overlie sediments with CaCO_3_ contents exceeding 80% dry weight, it is likely that our observation of calcification-driven CO_2_ emissions from this seagrass meadow applies to carbonate systems globally.

## DISCUSSION

### Progress toward realistic Blue Carbon accounting

Previous Blue Carbon syntheses have suggested carbonate seagrass meadows as strong carbon sinks, based largely on the assumption that much of the CaCO_3_ was allochthonous (*10*, *12*). The present study site in central Florida Bay is isolated from coral reefs and other calcifying ecosystems, meaning that all the CaCO_3_ buried here is produced in situ (*38*, *42*). Measured rates of lime mud production in Florida Bay suggest that Florida Bay exports carbonates rather than importing them (*42*). In addition, extensive mud banks throughout Florida Bay restrict horizontal exchange (*43*), further decreasing the chance of internal CaCO_3_ redistribution, as previously suggested (*12*). Therefore, our direct EC measurements, which reveal net annual emissions of 700 ± 660 μmol CO_2_ m^−2^ hour^−1^ (6.1 mol m^−2^ year^−1^), place this seagrass meadow as a clear net CO_2_ source to the atmosphere. Calcification explained essentially all the measured CO_2_ emissions and exceeded the OC sink by more than 300%, rather than offsetting 30 to 40% of OC burial as previous work indicated (*12*). This net CaCO_3_ burial is sustained by carbonate dissolution and reprecipitation, enhancing CaCO_3_ burial efficiency and ultimately shaping CO_2_ emissions. While OC burial does offset a small fraction (3.4%) of these emissions, an unrealistic 20-fold increase would be required for the site to become a net CO_2_ sink. The impact of TA production by net sulfate and Fe reduction is even smaller, offsetting ~1% of the calcification CO_2_ emissions. While our EC CO_2_ fluxes are representative of a full annual cycle, the relative IC, primary productivity, denitrification, etc., on net CO_2_ emissions vary with seasonal variations in temperature and salinity. The seasonal increase in afternoon CO_2_ emissions during warmer months (Fig. 1A) is consistent with the role of temperature in enhancing net CO_2_ release. This is likely a combined result of increased ecosystem calcification rates, along with the role that temperature plays in physically enhancing air-water gas transfer through convective forcing as documented previously at this site (*22*).

Our empirical approach, supported by micrometeorological and isotope geochemical tools, contributes fundamental evidence toward resolving one of the key uncertainties in Blue Carbon accounting (*13*, *18*). This approach is especially suitable for other ecosystems considered for Blue Carbon storage where hydrodynamic forcing is minimal, allowing measured air-water CO_2_ exchanges to be more reliably linked to benthic biogeochemical processing. However, a decoupling of benthic processes from air-water exchanges may occur when tidal exchanges are dominant, challenging this approach in more complex estuarine environments. Furthermore, our study considered a seagrass meadow with relatively low biomass and P limitation, but in seagrass meadows with increased P availability, greater standing biomass, enhanced primary production, or net carbonate dissolution (*35*), CO_2_ emissions could be decreased facilitating net C storage. Nevertheless, as more than a quarter of all Blue Carbon habitats also have high carbonate contents (>80% dry weight) (*12*), reliable carbon balancing such as this study will help identify cases of “surprise” CO_2_ emissions (*44*). Our study motivates a more rigorous (re-)assessment of the coastal carbon balance, which is essential to protect public and private sectors’ initiatives and investments in ocean-based mitigation of climate change as an accountable contribution to the COP21 Paris agreement.

## MATERIALS AND METHODS

### Site description and sample collection

All collection of discrete samples was carried out in November 2019 at a well-characterized and shallow site (~1 m depth, 25°1.718′ N, 80°40.736′ W) near Bob Allen Keys in central Florida Bay, one of the largest seagrass-dominated estuaries in the world. Sediments consist of 70 to 90% of carbonate minerals (>90% for Bob Allen Keys) (*21*), mostly aragonite and high Mg calcite (*26*), with relatively low iron content (*28*, *45*), and only trace amounts of silicate minerals (*46*). The site is a sparse-medium density seagrass meadow dominated by *Thalassia testudinum* (turtle grass) with occasional occurrences of *Halodule wrightii* and sparsely distributed macroalgae. Low phosphorus availability limits the development of dense seagrass meadows in this part of Florida Bay (*47*). We characterized the species composition and relative abundance with surveys occuring six times per year for the period 2000–2021 along a permanently marked 50-m transect. During each survey, all conspicuous taxa in 10 randomly placed 0.25 m^2^ sampling quadrats along the transect were identified and assigned a Braun-Blanquet cover-abundance score (*48*, *49*). We transformed the cover and abundance scores to percent cover (defined as the fraction of the bottom obscured by the taxon of interest when viewed from above) assuming the midpoint of each cover range for each cover-abundance score. Over the 112 surveys conducted over the 22-year period of record, *T. testudinum* was the dominant species, averaging 18.6 ± 1.0% cover (Table 1). The only other seagrass species encountered was *H. wrightii*, which was detected in 18 of the surveys with a low period-of-record average abundance of 0.05 ± 0.01% cover (Table 1). Calcifying and noncalcifying macroalgae were also present, but at lower percent cover than the dominant *T. testudinum.* Of these macroalgae, *Penicillus* sp. was the most often encountered and was observed on 100 of the 112 total transects (at an average of 0.8 ± 0.1% cover). The macroalgae *Batophora* sp. had the highest average percent cover of 2.8 ± 0.7% but was present less frequently.

**Table 1.** Abundance, expressed as percent cover, for the conspicuous benthic macrophytes and sponges encountered on the 112 surveys of a 50-m transect sampled nominally six times per year from 2000 to 2021.

**Taxon**	**Mean ± 1 SE**	**No. of occurrences**	**Minimum**	**Maximum**
Seagrasses				
*T. testudinum*	18.6 ± 1.0	112	2.2	57.5
*Halodule wrightii*	0.05 ± 0.01	18	0	1
Macroalgae				
*Chlorophyta*				
*Penicillus* sp.	0.8 ± 0.1	100	0	3.8
*Rhipocephalus* sp.	0.002 ± 0.001	3	0	0.125
*Acetabularia* sp.	0.002 ± 0.001	2	0	0.125
*Neomeris* sp.	0.05 ± 0.04	3	0	3.75
*Cymopolia* sp.	0.07 ± 0.01	54	0	0.5
*Batophora* sp.	2.8 ± 0.7	76	0	57.5
*Dasycladus* sp.	0.2 ± 0.1	11	0	15
Other Chlorophyta	1.4 ± 0.3	49	0	15
*Rhodophyta*				
Drift algae	0.3 ± 0.2	10	0	11.25
Other Rhodophyta	0.2 ± 0.1	9	0	11.25
*Phaeophyta*				
*Sargassum* sp.	0.014 ± 0.007	5	0	0.5
Other *Phaeophyta*	0.4 ± 0.2	7	0	21.75
Sponges	0.12 ± 0.02	46	0	1.1

**Fig. 5.**
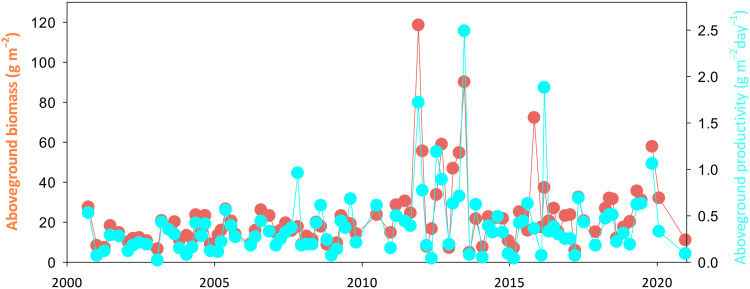
Time series of aboveground biomass and aboveground net primary productivity of the dominant seagrass *T. testudinum* at the EC tower site for the period of record demonstrating seasonality but no long-term monotonic trends.

Both the aboveground biomass and the aboveground net primary productivity of *T. testudinum* were seasonal at the site and showed no long-tern monotonic trends. We measured productivity using the leaf-mark technique (*50*) in six 10 cm–by–20 cm quadrats six times per year from the period 2000–2021. Aboveground biomass was assessed as the mass of green leaves in the productivity quadrats and averaged 22.2 ± 1.8 g (dry weight) m^−2^ (±1 SE, *n* = 96; Fig. 5), and aboveground net primary productivity averaged 0.40 ± 0.04 g (dry weight) m^−2^ day^−1^. For each biomass sample, we dried the compounded leaves and ground them to a fine powder for analysis of C content using an elemental analyzer; *T. testudinum* leaves averaged 38.3 ± 0.2% C (*n* = 125) over the 22 years of sampling. The C stocks in the aboveground biomass of *T. testudinum* (which comprised the vast majority of the total aboveground biomass of macrophytes at the site) averaged 0.085 Mg C ha^−1^, falling below the global median C stock (*1*) of 0.264 Mg C ha^−1^. This low seagrass biomass is typical of this region of Florida Bay (*51*) and representative of the seagrass meadows of the footprint of our EC tower. Our average percent cover of *T. testudinum* of 18.6% was similar to the percent cover detected in the Florida Fish and Wildlife Research Institute’s Fisheries Habitat Assessment Program (FHAP) for Florida Bay that has been assessing percent cover using the Braun-Blanquet technique twice per year since 1995 (*52*). The FHAP program uses a stratified-random procedure to distribute 30 observation points evenly across basins of Florida Bay for each survey. In the 36-km^2^ basin immediately to the north of our study site, *T. testudinum* averaged 12.4 ± 0.4% cover for 1011 observations over the period of record. In the 20-km^2^ basin immediately to the east of our study site, *T. testudinum* cover averaged 14.3 ± 0.4%. No FHAP data exist for the basin to the west of our study site, but it has a visually similar characteristic seagrass meadow as the site and the other two basins.

We assessed net benthic metabolic rates at three locations in close proximity within this seagrass meadow, representing regions of (i) high seagrass aboveground biomass (HD), (ii) low seagrass density (LD), and (iii) bare sediment (B). We collected samples at these three sites to characterize as fully as possible the seagrass meadow that occupies the EC footprint. Quadruplicate cores containing approximately 10 cm of sediment were collected for a continuous-flow incubation in which net sediment-water fluxes were directly measured using cores that either included or excluded living seagrass biomass. This steady-state incubation does not represent net ecosystem metabolism because it was conducted in the dark. Rather, the intent was to create net conditions representative of night-time heterotrophy when denitrification is expected to be greatest (*31*), thereby providing a conservative estimate for the maximum anaerobic alkalinity that could be expected, across a realistic range in seagrass density. Separately, duplicate 30- to 50-cm sediment cores were collected at HD and LD sites for pore-water and solid-phase analysis.

### Sediment and pore-water sampling and analysis

For solid-phase characterization, we collected and froze 50-cm-long acrylic cores at both HD and LD sites, which were thawed and subsampled at 5-cm intervals within 1 week of sampling. Sediment compaction due to inserting the tubes was not visible or indicated by the vertical bulk density profiles. Following transport on dry ice back to the laboratory, sediment was freeze-dried, ground, and microwave-digested in a solution of 5-ml concentrated HNO_3_ and 2-ml concentrated HCl before trace-metal analysis described below. Frozen sediment was freeze-dried and analyzed for δ^13^C of the inorganic carbonate fraction (δ^13^C_PIC_) or preserved with 25% Zn(oAc)_2_ for the analysis of CRS (a measure for S^o^ and FeS_2_) and AVS (a proxy for H_2_S and FeS). The AVS and CRS fractions were extracted from the sediments according to a two-step distillation procedure (*53*), as described in (*54*, *55*). Duplicate acrylic cores of 25 to 35 cm length were furthermore collected from HD and LD sites, and pore water was retrieved at 5-cm-depth resolution using Rhizons (0.12-μm-diameter filtration) on the same day as sampling. Pore water was preserved with HgCl_2_, stored cool and dark, and analyzed for the concentration and stable carbon isotope composition of DIC (δ^13^C_DIC_) (*56*) using a Thermo Finnigan MAT 253 gas mass spectrometer coupled to GasBench II. Pore-water aliquots preserved with 5% Zn(oAc)_2_ were used for spectrophotometric measurement of the total dissolved sulfide concentration (*57*) using an Analytik Jena AG, Specord 40 photometer (*56*). δ^13^C measurements of DIC and CaCO_3_ were carried out via isotope ration monitoring mass spectrometry as described by (*58*) and (*59*), respectively. The resulting per mil values are equivalent to mUr [milli-Urey (*60*)]. Isotope ratio measurements are given with respect to the international Vienna Pee Dee Belemnite (VPDB) standard.

### Sediment trace metal analysis

Sediment solids were sampled and frozen, as described above, before trace metal analysis. Type I reagent-grade water (>18.2 megohm·cm) for trace metal analysis was obtained from an ultrapure water system consisting of an Elix 3 module (Merck Millipore), a Milli-Q element module (Merck Millipore), and a Q-POD element (Merck Millipore). Analytical grade HNO_3_ (65%, w/w; Fisher Scientific GmbH) and analytical grade HCl (30%, w/w, Carl Roth GmbH + Co. KG) were further purified by double subboiling in perfluoroalkoxy-polymer (PFA)–subboiling stills (DST-4000 and DST-1000, Savillex). Sediment was freeze-dried (Beta 1-8, Christ Gefriertrocknungsanlagen), ground (PM 400, Retsch), and microwave-digested (MARS 5 Xpress, CEM Corp.) in a solution of 5-ml concentrated HNO_3_ and 2-ml concentrated HCl before trace-metal analysis (*61*) by inductively coupled plasma–tandem mass spectrometry (ICP-MS/MS) (Agilent 8800, Agilent Technologies). Digestion took place at 180°C for 300 min using 35-ml precleaned TFM digestion vessels. ICP-MS/MS was coupled to an ESI SC-4 DX FAST autosampler (Elemental Scientific) equipped with a discrete sampling system with a loop volume of 1.5 ml. Selection of the analyzed isotope, as well as cell gas mode, was based on the achieved sensitivity and occurrence of spectral interferences (isobaric and polyatomic interferences): ^24^Mg (H_2_ mode), ^40^Ca (H_2_ mode), ^56^Fe (H_2_ mode), ^88^Sr (He mode), and ^95^Mo (O_2_ mass shift mode). The instrument was optimized on a daily basis using a tune solution containing Li, Y, Ce, and Tl (10 μg liter^−1^). Quantification was performed on the basis of external calibration, covering a concentration range from 0.1 to 100 μg liter^−1^ for all analytes and from 10 to 10,000 μg liter^−1^ for Ca. Solutions were prepared on a daily basis from custom-made multielement standards (Inorganic Ventures). Multielement data were processed using MassHunter versions 4.4 (Agilent Technologies) and a custom-written Excel spreadsheet. The significant number of digits of mass fractions is given according to GUM (Guide to the Expression of Uncertainty in Measurement) and EURACHEM guidelines, whereby the uncertainty determines the significant number of digits to be presented with the value. A detailed description of the trace metal determination can be found in the literature (*61*).

### Air-water CO_2_ fluxes

Air-water CO_2_ fluxes were continuously monitored by atmospheric EC such that the flux footprint captured the spatial domain of sediment and pore-water sampling. CO_2_ fluxes were calculated in EddyPro (LI-COR Biosciences) at 30-min intervals from continuous high-frequency (10 Hz) data, and these 30-min fluxes were averaged to generate the daily or hourly fluxes presented in Fig. 1 (B and C). Briefly, the high-frequency data were processed in EddyPro, which effectively handled tilt correction, time-lag compensation, WPL (Webb) correction, high/low pass filtering, and other factors. The 30-min averages produced by EddyPro were further screened by a variety of steps attempting to limit results to when the air-water flux footprint was representative only of the seagrasses under study to exclude conditions of nonstationarity or when the gas analyzer optical path was dirty. Detailed methods for EC measurements and associated data screening procedures are identical to those applied previously (*22*).

### Measured sediment-water flux

Continuous flow experiments with intact sediment cores were used to determine fluxes of dissolved gases across the sediment-water interface (*33*). Quadruplicate cores of 10 cm deep, 6.4 cm diameter with overlying water were collected at site B, and at two separate subsites for HD and LD, one set containing aboveground seagrass biomass, and the other omitting biomass for 20 cores total. These cores were incubated under continuous flowing water at a flow rate of 1.5 ml/min with aerated site water at room temperature (21.6°C) and in the dark. Cores were preincubated for 24 hours to allow the system to reach steady state (*33*). This setup prevented changes between net photosynthesis and respiration driven by light-to-dark transitions, a necessary step to prevent the formation of bubbles that can affect dissolved gas measurements and ensure that calculated N_2_ and DOC fluxes were representative of steady-state conditions (*62*). Inflow and outflow samples were collected five times over the next 48 hours. Sediment-water fluxes of N_2_ (positive flux is net denitrification in excess of nitrogen fixation) and DOC (Fig. 1) were determined from the difference between each outflow and inflow of site reference water, flow rate, and surface area (*62*). Samples for N_2_ were preserved with 100 μl of saturated ZnCl_2_. N_2_ was measured by membrane inlet mass spectrometry. Samples for DOC analysis were filtered and acidified with HCl and frozen before analysis. DOC concentration was measured on a modified OI Analytical Aurora Model 1030 TOC analyzer using a high-temperature combustion method, coupled to a Delta V Plus isotope ratio mass spectrometer for δ^13^C measurement (*63*). Samples were acidified to PH 2 with 85% phosphoric acid and sparged using ultrahigh-purity argon. DOC values were blank-corrected with ultrapure Milli-Q water (18.2-megohm resistivity) and calibrated with caffeine standards, ranging from 83 to 3320 μmol C liter^−1^. Both caffeine and sucrose standards of known carbon isotopic composition obtained from the International Atomic Energy Agency (IAEA C6) were used to express stable isotope values in the delta notation “δ,” where the ratio (*R*) of ^13^C to ^12^C for an unknown sample is calibrated against a known standard

$$\delta^{13}C\;(‰)=[(R_{\text{sample}}/R_{\text{standard}}-1)\times 1000]$$

The values are reported as per mil relative to VPDB. The precision of the analysis for [DOC] was ±16 μmol C liter^−1^ and ±0.2‰ for δ^13^C-DOC. By convention, positive fluxes represent a release from the sediment to the overlying water and negative fluxes are movement from the water column to the sediment.

### Modeled sediment-water flux

Direct measurements of sediment-water fluxes were augmented by advective-diffusive modeling in PROFILE (*64*) for H_2_S and DIC. We assumed no irrigation and estimated biodiffusivity in the upper 15 cm of sediment at 1 and 3 cm^2^ hour^−1^ (0.00028 and 0.00083 cm^2^ s^−1^) at the LD and HD sites, respectively, representing seagrass O_2_ pumping into the rhizosphere. We arrived at these parameterizations through iterative calculation runs attempting to match model output DIC concentration profiles and vertical flux with measured DIC profiles and fluxes. Molecular diffusivity was calculated at in situ temperature and salinity using the “marelac” package (*65*) in R. Boundary conditions at the top were set by the surface water concentration and at the bottom of the model domain by a diffusive flux calculated from the gradient across the bottom two concentration measurements.

### Budget creation

Last, we constructed a carbon and alkalinity budget, combining literature SARs (*37*, *38*) with our solid-phase results to estimate net accumulation of CaCO_3_, and reduced sulfur and iron species. Using the stoichiometry outlined in Table 2, these burial rates were converted into net fluxes of DIC and TA, which were, in turn, used to calculate the CO_2_ production or consumption due to each biogeochemical process. The ultimate goal of this exercise was to compare budgeted excess CO_2_ with the excess CO_2_ that would be required to sustain the CO_2_ fluxes that were directly observed in this study.

**Table 2.** Selection of generalized biogeochemical reaction pathways affecting TA and DIC. Asterisk indicates that the value is not always 0 (see Budget creation section).

**Process**	**Generalized reaction**	**ΔTA**	**ΔDIC**	**Inferred from**
Primary production	CO_2_ + H_3_PO_4_ + HNO_3_ → OM + O_2_	0*	−1	O_2_, nutrient flux
Sulfate reduction + burial as 50% FeS_2_ and 50% S^0^	$4\;\text{OM}+2{\text{SO}}_{4}^{2-}+2H^{+}\to 4{\text{CO}}_{2}+2H_{2}S$	+2	+1	CRS
	+			
	0.5 × [FeS + H_2_S → FeS_2_ + H_2_] 0.5× [O_2_ + 2H^+^ + H_2_S → S^o^ + 2H_2_O]	$\left[1+\frac{0+2}{2}\right]$	$\left[1+\frac{0+0}{2}\right]$	
Fe reduction	OM + 2Fe_2_O_3_ + 8H^+^ → 2CO_2_ + 4Fe^2+^	+8	+2	Fe—CRS
Canonical denitrification	OM + 0.8 HNO_3_ → CO_2_ + 0.4N_2_ + NH_3_ + H_3_PO_4_	+0.8	+1	N_2_ flux
CaCO_3_ precipitation	${\text{Ca}}^{2+}+2{\text{HCO}}_{3}^{-}\to {\text{CaCO}}_{3}+{\text{CO}}_{2}+H_{2}O$	−2	−1	Range of literature CaCO_3_ accumulation rates

We assumed a range in literature carbonate accumulation rates (*37*, *38*) of 1.9 to 1042 g m^−2^ year^−1^ or 0.019 to 10.4 mol CaCO_3_ m^−2^ year^−1^. According to our solid-phase analysis, this sediment is 97.5% IC, 2.1% OC, 0.3% CRS (representing FeS_2_ and S^0^), and approximately 0.5% “free” Fe (calculated as the difference between CRS and total Fe). The net impact of production and burial of IC and OC on TA and DIC is shown in Table 2. The role of sulfate reduction is rather more complicated, as the sulfide produced by sulfate reduction (ΔTA:ΔDIC = 1:1) may or may not react with various phases of Fe, with various effects (*66*) on TA. Therefore, we take a simplifying approach of assuming that CRS burial occurs in equal proportions as FeS_2_ (ΔTA = 0) and S^0^ (ΔTA = 2) such that the net burial of reduced sulfide results in the production of TA and DIC in a ratio of 2:1 (Table 2). The net formation of free Fe produces TA and DIC in a ratio (*66*) of 8:2. Our modeling and experimental results demonstrate that N cycling is tightly internally recycled such that the net rates are indistinguishable from zero, meaning that the impact of net denitrification on sediment TA release can be neglected from this model. Similarly, because NO*_x_* flux was balanced very closely by NH_4_ flux (not shown in figures), we can assume that the net effect of net ecosystem production on TA was small (as indicated by the asterisk in Table 2). These molar equivalents were combined with literature SARs to estimate the change in water-column TA and DIC due to each of the abovementioned processes [IC burial, OC burial, net SO_4_ reduction (CRS), and Fe reduction].

We calculated the change in surface water CO_2_ concentration ($\frac{\text{∆}{\text{CO}}_{2}}{{\text{CO}}_{2}}$) for each process using the above TA and DIC anomalies (*X* and *Y*; Table 2) and buffer factors (*67*) for TA (γ_TA_) and DIC (γ_DIC_): $\frac{\text{∆}{\text{CO}}_{2}}{{\text{CO}}_{2}}=\frac{\text{SAR}\times X\;}{\gamma_{\text{DIC}}}+\frac{\text{SAR}\times Y}{\gamma_{\text{TA}}}$. Subsequently, the CO_2_ concentration (μmol kg^−1^) resulting from this $\frac{\text{∆}{\text{CO}}_{2}}{{\text{CO}}_{2}}$ was then calculated using the “seacarb” package (*68*) in RStudio. Initial conditions were set according to direct measurements in the water column (TA, 2596 μmol kg^−1^; DIC, 2295 μmol kg^−1^; salinity, 38; temperature, 26°C). The excess CO_2_ (μmol kg^−1^) shown in Fig. 4 is the difference between initial CO_2_ and the CO_2_ concentration after sediment TA/DIC exchange. Water currents at this site are very minor (*22*), allowing us to exclude lateral exchanges from this simple model. However, an important implication of this model is that any net TA and DIC production/consumption is compensated by import/export with the coastal ocean, albeit over longer time scales.

## References

[1] J. W. Fourqurean, C. M. Duarte, H. Kennedy, N. Marba, M. Holmer, M. A. Mateo, E. T. Apostolaki, G. A. Kendrick, D. Krause-Jensen, K. J. McGlathery, O. Serrano, Seagrass ecosystems as a globally significant carbon stock. Nat. Geosci. 5, 505–509 (2012).

[2] T. Tokoro, K. Watanabe, K. Tada, T. Kuwae, Air–water CO_2_ flux in shallow coastal waters: Theory, methods, and empirical studies, in *Blue Carbon in Shallow Coastal Ecosystems: Carbon Dynamics, Policy, and Implementation*, T. Kuwae, M. Hori, Eds. (Springer, 2019), pp. 153–184.

[3] C. M. Duarte, J. J. Middelburg, N. Caraco, Major role of marine vegetation on the oceanic carbon cycle. Biogeosciences 2, 1–8 (2005).

[4] J.-P. Gattuso, A. K. Magnan, L. Bopp, W. W. L. L. Cheung, C. M. Duarte, J. Hinkel, E. Mcleod, F. Micheli, A. Oschlies, P. Williamson, R. Billé, V. I. Chalastani, R. D. Gates, J. O. Irisson, J. J. Middelburg, H.-O. Pörtner, G. H. Rau, Ocean solutions to address climate change and its effects on marine ecosystems. Front. Mar. Sci. 5, 337 (2018).

[5] J. Howard, A. Sutton-Grier, D. Herr, J. Kleypas, E. Landis, E. Mcleod, E. Pidgeon, S. Simpson, Clarifying the role of coastal and marine systems in climate mitigation. Front. Ecol. Environ. 15, 42–50 (2017).

[6] H. Kennedy, J. Beggins, C. M. Duarte, J. W. Fourqurean, M. Holmer, N. Marbá, J. J. Middelburg, Seagrass sediments as a global carbon sink: Isotopic constraints. Glob. Biogeochem. Cycles 24, GB4026 (2010).

[7] B. A. Needelman, I. M. Emmer, S. Emmett-Mattox, S. Crooks, J. P. Megonigal, D. Myers, M. P. J. Oreska, K. McGlathery, The science and policy of the verified carbon standard methodology for tidal wetland and seagrass restoration. Estuar. Coasts 41, 2159–2171 (2018).

[8] J. L. Howard, J. C. Creed, M. V. P. Aguiar, J. W. Fouqurean, CO_2_ released by carbonate sediment production in some coastal areas may offset the benefits of seagrass “Blue Carbon” storage. Limnol. Oceanogr. 63, 160–172 (2018).

[9] P. I. Macreadie, O. Serrano, D. T. Maher, C. M. Duarte, J. Beardall, Addressing calcium carbonate cycling in blue carbon accounting. Limnol. Oceanogr. Lett. 2, 195–201 (2017).

[10] I. Mazarrasa, N. Marbà, C. E. Lovelock, O. Serrano, P. S. Lavery, J. W. Fourqurean, H. Kennedy, M. A. Mateo, D. Krause-Jensen, A. D. L. Steven, C. M. Duarte, Seagrass meadows as a globally significant carbonate reservoir. Biogeosciences 12, 4993–5003 (2015).

[11] X. Hu, W.-J. Cai, An assessment of ocean margin anaerobic processes on oceanic alkalinity budget. Glob. Biogeochem. Cycles 25, GB3003 (2011).

[12] V. Saderne, N. R. Geraldi, P. I. Macreadie, D. T. Maher, J. J. Middelburg, O. Serrano, H. Almahasheer, A. Arias-Ortiz, M. Cusack, B. D. Eyre, J. W. Fourqurean, H. Kennedy, D. Krause-Jensen, T. Kuwae, P. S. Lavery, C. E. Lovelock, N. Marba, P. Masqué, M. A. Mateo, I. Mazarrasa, K. J. M. Glathery, M. P. J. Oreska, C. J. Sanders, I. R. Santos, J. M. Smoak, T. Tanaya, K. Watanabe, C. M. Duarte, Role of carbonate burial in Blue Carbon budgets. Nat. Commun. 10, 1106 (2019). 30846688 10.1038/s41467-019-08842-6 PMC6405941

[13] P. I. Macreadie, A. Anton, J. A. Raven, N. Beaumont, R. M. Connolly, D. A. Friess, J. J. Kelleway, H. Kennedy, T. Kuwae, P. S. Lavery, C. E. Lovelock, D. A. Smale, E. T. Apostolaki, T. B. Atwood, J. Baldock, T. S. Bianchi, G. L. Chmura, B. D. Eyre, J. W. Fourqurean, J. M. Hall-Spencer, M. Huxham, I. E. Hendriks, D. Krause-Jensen, D. Laffoley, T. Luisetti, N. Marbà, P. Masque, K. J. McGlathery, J. P. Megonigal, D. Murdiyarso, B. D. Russell, R. Santos, O. Serrano, B. R. Silliman, K. Watanabe, C. M. Duarte, The future of Blue Carbon science. Nat. Commun. 10, 3998 (2019). 31488846 10.1038/s41467-019-11693-w PMC6728345

[14] N. A. O’Mara, J. P. Dunne, Hot spots of carbon and alkalinity cycling in the coastal oceans. Sci. Rep. 9, 4434 (2019). 30872724 10.1038/s41598-019-41064-w PMC6418168

[15] V. Saderne, M. Fusi, T. Thomson, A. Dunne, F. Mahmud, F. Roth, S. Carvalho, C. M. Duarte, Total alkalinity production in a mangrove ecosystem reveals an overlooked Blue Carbon component. Limnol. Oceanogr. Lett. 6, 61–67 (2021).

[16] M. Koch, G. Bowes, C. Ross, X. H. Zhang, Climate change and ocean acidification effects on seagrasses and marine macroalgae. Glob. Chang. Biol. 19, 103–132 (2013). 23504724 10.1111/j.1365-2486.2012.02791.x

[17] E. McLeod, G. L. Chmura, S. Bouillon, R. Salm, M. Björk, C. M. Duarte, C. E. Lovelock, W. H. Schlesinger, B. R. Silliman, A blueprint for blue carbon: Toward an improved understanding of the role of vegetated coastal habitats in sequestering CO_2_. Front. Ecol. Environ. 9, 552–560 (2011).

[18] N. L. Bindoff, W. W. L. Cheung, J. G. Kairo, J. Arístegui, V. A. Guinder, R. Hallberg, N. Hilmi, N. Jiao, M. S. Karim, L. Levin, S. O’Donoghue, S. R. Purca Cuicapusa, B. Rinkevich, T. Suga, A. Tagliabue, P. Williamson, Changing ocean, marine ecosystems, and dependent communities, in *IPCC Special Report on the Ocean and Cryosphere in a Changing Climate*, H.-O. Pörtner, D. C. Roberts, V. Masson-Delmotte, P. Zhai, M. Tignor, E. Poloczanska, K. Mintenbeck, A. Alegría, M. Nicolai, A. Okem, J. Petzold, B. Rama, N. M. Weyer, Eds. (2019) [in press].

[19] S. Thomas, Blue carbon: Knowledge gaps, critical issues, and novel approaches. Ecol. Econ. 107, 22–38 (2014).

[20] S. Tramel, The road through Paris: Climate change, carbon, and the political dynamics of convergence. Globalizations 13, 960–969 (2016).

[21] J. W. Fourqurean, G. A. Kendrick, L. S. Collins, R. M. Chambers, M. A. Vanderklift, Carbon, nitrogen and phosphorus storage in subtropical seagrass meadows: Examples from Florida Bay and Shark Bay. Mar. Freshw. Res. 63, 967–983 (2012).

[22] B. R. Van Dam, C. C. Lopes, P. Polsenaere, R. M. Price, A. Rutgersson, J. W. Fourqurean, Water temperature control on CO_2_ flux and evaporation over a subtropical seagrass meadow revealed by atmospheric eddy covariance. Limnol. Oceanogr. 66, 510–527 (2020).

[23] F. J. Millero, W. Hiscock, F. Huang, M. Roche, Seasonal variation of the carbonate system in Florida Bay. Bull. Mar. Sci. 68, 101–123 (2001).

[24] J.-Z. Zhang, C. J. Fischer, Carbon dynamics of Florida Bay: Spatiotemporal patterns and biological control. Environ. Sci. Technol. 48, 9161–9169 (2014). 25020272 10.1021/es500510z

[25] M. P. J. Oreska, K. J. McGlathery, L. R. Aoki, A. C. Berger, P. Berg, L. Mullins, The greenhouse gas offset potential from seagrass restoration. Sci. Rep. 10, 7325 (2020). 32355280 10.1038/s41598-020-64094-1 PMC7193639

[26] L. M. Walter, T. C. W. Ku, K. Muehlenbachs, W. P. Patterson, L. Bonnell, Controls on the δ13C of dissolved inorganic carbon in marine pore waters: An integrated case study of isotope exchange during syndepositional recrystallization of biogenic carbonate sediments (South Florida Platform, USA). Deep. Res. Part II Top. Stud. Oceanogr. 54, 1163–1200 (2007).

[27] X. Hu, D. J. Burdige, Enriched stable carbon isotopes in the pore waters of carbonate sediments dominated by seagrasses: Evidence for coupled carbonate dissolution and reprecipitation. Geochim. Cosmochim. Acta 71, 129–144 (2007).

[28] R. M. Chambers, J. W. Fourqurean, S. A. Macko, R. Hoppenot, Biogeochemical effects of iron availability on primary producers in a shallow marine carbonate environment. Limnol. Oceanogr. 46, 1278–1286 (2001).

[29] M. Holmer, H. Hasler-Sheetal, Sulfide intrusion in seagrasses assessed by stable sulfur isotopes—A synthesis of current results. Front. Mar. Sci. 1, 64 (2014).

[30] H. Hasler-Sheetal, M. Holmer, Sulfide intrusion and detoxification in the seagrass *Zostera marina*. PLOS ONE 10, e0129136 (2015). 26030258 10.1371/journal.pone.0129136 PMC4452231

[31] B. D. Eyre, D. Maher, J. M. Oakes, D. V. Erler, T. M. Glasby, Differences in benthic metabolism, nutrient fluxes, and denitrification in *Caulerpa taxifolia* communities compared to uninvaded bare sediment and seagrass (*Zostera capricorni*) habitats. Limnol. Oceanogr. 56, 1737–1750 (2011).

[32] B. D. Eyre, A. J. P. Ferguson, Comparison of carbon production and decomposition, benthic nutrient fluxes and denitrification in seagrass, phytoplankton, benthic microalgae- and macroalgae-dominated warm-temperate Australian lagoons. Mar. Ecol. Prog. Ser. 229, 43–59 (2002).

[33] W. S. Gardner, M. J. McCarthy, Nitrogen dynamics at the sediment-water interface in shallow, sub-tropical Florida Bay: Why denitrification efficiency may decrease with increased eutrophication. Biogeochemistry 95, 185–198 (2009).

[34] B. D. Eyre, I. R. Santos, D. T. Maher, Seasonal, daily and diel N_2_ effluxes in permeable carbonate sediments. Biogeosciences 10, 2601–2615 (2013).

[35] M. A. Zeller, B. R. Van Dam, C. Lopes, J. S. Kominoski, Carbonate-associated organic matter is a detectable dissolved organic matter source in a subtropical seagrass meadow. Front. Mar. Sci. 7, 580284 (2020).

[36] M. Long, P. Berg, J. Falter, Seagrass metabolism across a productivity gradient using the eddy covariance, Eulerian control volume, and biomass addition techniques. J. Geophys. Res. Ocean. 120, 2676–2700 (2015).

[37] T. A. Frankovich, J. C. Zieman, Total epiphyte and epiphytic carbonate production on Thalassia testudinum across Florida Bay. Bull. Mar. Sci. 54, 679–695 (1994).

[38] D. W. J. Bosence, Biogenic carbonate production in florida bay. Bull. Mar. Sci. 44, 419–433 (1989).

[39] A. R. Armitage, J. W. Fourqurean, Carbon storage in seagrass soils: Long-term nutrient history exceeds the effects of near-term nutrient enrichment. Biogeosciences 13, 313–321 (2016).

[40] M. Gullström, L. D. Lyimo, M. Dahl, and others, Blue carbon storage in tropical seagrass meadows relates to carbonate stock dynamics, plant–sediment processes, and landscape context: Insights from the western Indian ocean. Ecosystems 21, 551–566 (2018).

[41] C. J. Sanders, D. T. Maher, J. M. Smoak, B. D. Eyre, Large variability in organic carbon and CaCO_3_ burial in seagrass meadows: A case study from three Australian estuaries. Mar. Ecol. Prog. Ser. 616, 211–218 (2019).

[42] K. W. Stockman, R. N. Ginsburg, E. A. Shinn, The production of lime mud by algae in south Florida. J. Sediment. Petrol. 37, 633–648 (1967).

[43] R. N. Ginsburg, Environmental relationships of grain size and constituent particles in some south Florida carbonate sediments. Am. Assoc. Pet. Geol. 40, 2384–2427 (1956).

[44] A. Akhand, K. Watanabe, A. Chanda, T. Tokoro, K. Chakraborty, H. Moki, T. Tanaya, J. Ghosh, T. Kuwae, Lateral carbon fluxes and CO_2_ evasion from a subtropical mangrove-seagrass-coral continuum. Sci. Total Environ. 752, 142190 (2021). 33207513 10.1016/j.scitotenv.2020.142190

[45] S. Ruiz-Halpern, S. A. Macko, J. W. Fourqurean, The effects of manipulation of sedimentary iron and organic matter on sediment biogeochemistry and seagrasses in a subtropical carbonate environment. Biogeochemistry 87, 113–126 (2008).

[46] T. C. W. Ku, L. M. Walter, M. L. Coleman, R. E. Blake, A. M. Martini, Coupling between sulfur recycling and syndepositional carbonate dissolution: Evidence from oxygen and sulfur isotope composition of pore water sulfate, South Florida Platform, U.S.A. Geochim. Cosmochim. Acta 63, 2529–2546 (1999).

[47] J. W. Fourqurean, J. C. Zieman, G. V. N. Powell, Phosphorus limitation of primary production in Florida Bay: Evidence from C:N:P ratios of the dominant seagrass *Thalassia testudinum*. Limnol. Oceanogr. 37, 162–171 (1992).

[48] W. J. Kenworthy, M. J. Durako, S. M. R. Fatemy, H. Valavi, G. W. Thayer, Ecology of seagrasses in northeastern Saudi Arabia one year after the Gulf War oil spill. Mar. Pollut. Bull. 27, 213–222 (1993).

[49] J. W. Fourqurean, A. Willsie, C. D. Rose, L. M. Rutten, Spatial and temporal pattern in seagrass community composition and productivity in south Florida. Mar. Biol. 138, 341–354 (2001).

[50] J. C. Zieman, Methods for the study of the growth and production of turtle grass, Thalassia testudinum König. Aquaculture 4, 139–143 (1974).

[51] J. C. Zieman, J. W. Fourqurean, R. L. Iverson, Distribution, abundance and productivity of seagrasses and macroalgae in Florida Bay. Bull. Mar. Sci. 44, 292–311 (1989).

[52] M. J. Durako, M. O. Hall, M. Merello, Patterns of change in the seagrass dominated Florida Bay hydroscape, in *The Everglades, Florida Bay, and the Coral Reefs of the Florida Keys*, J. W. Porter, K. G. Porter, Eds. (CRC Press, 2002), pp. 523–538.

[53] H. Fossing, B. B. Jørgensen, Measurement of bacterial sulfate reduction in sediments: Evaluation of a single-step chromium reduction method. Biogeochemistry 8, 205–222 (1989).

[54] S. L. Seibert, M. E. Böttcher, F. Schubert, T. Pollmann, L. Giani, S. Tsukamoto, M. Frechen, H. Freund, H. Waska, H. Simon, T. Holt, J. Greskowiak, G. Massmann, Iron sulfide formation in young and rapidly-deposited permeable sands at the land-sea transition zone. Sci. Total Environ. 649, 264–283 (2019). 30173034 10.1016/j.scitotenv.2018.08.278

[55] F. Koebsch, M. Winkel, S. Liebner, B. Liu, J. Westphal, I. Schmiedinger, A. Spitzy, M. Gehre, G. Jurasinski, S. Köhler, V. R. Unger, M. Koch, T. Sachs, M. E. Böttcher, Sulfate deprivation triggers high methane production in a disturbed and rewetted coastal peatland. Biogeosciences 16, 1937–1953 (2019).

[56] Z. Wu, B. Liu, P. Escher, N. Kowalski, M. E. Böttcher, Carbon diagenesis in different sedimentary environments of the subtropical Beibu Gulf, South China Sea. J. Mar. Syst. 186, 68–84 (2018).

[57] J. D. Cline, Spectrophotometric determination of hydrogen sulfide in natural WATERS1. Limnol. Oceanogr. 14, 454–458 (1969).

[58] V. Winde, M. E. Böttcher, P. Escher, P. Böning, M. Beck, G. Liebezeit, B. Schneider, Tidal and spatial variations of DI^13^C and aquatic chemistry in a temperate tidal basin during winter time. J. Mar. Syst. 129, 394–402 (2014).

[59] M. E. Böttcher, N. Neubert, P. Escher, K. von Allmen, E. Samankassou, T. F. Nägler, Multi-isotope (Ba, C, O) partitioning during experimental carbonatization of a hyperalkaline solution. CdE 78, 241–247 (2018).

[60] W. A. Brand, T. B. Coplen, Stable isotope deltas: Tiny, yet robust signatures in nature. Isot. Environ. Health Stud. 48, 393–409 (2012). 10.1080/10256016.2012.666977 22462621

[61] T. Zimmermann, M. von der Au, A. Reese, O. Klein, L. Hildebrandt, D. Pröfrock, Substituting HF by HBF4—An optimized digestion method for multi-elemental sediment analysisviaICP-MS/MS. Anal. Methods 12, 3778–3787 (2020). 32706011 10.1039/d0ay01049a

[62] A. R. Smyth, S. P. Thompson, K. N. Siporin, W. S. Gardner, M. J. McCarthy, M. F. Piehler, Assessing nitrogen dynamics throughout the estuarine landscape. Estuar. Coasts 36, 44–55 (2013).

[63] K. Lalonde, P. Middlestead, Y. Gélinas, Automation of ^13^C/^12^C ratio measurement for freshwater and seawater DOC using high temperature combustion. Limnol. Oceanogr. Methods 12, 816–829 (2014).

[64] P. Berg, R. Nils, S. Rysgaard, Interpretation of measured concentration profiles in sediment pore water. Limnol. Oceanogr. 43, 1500–1510 (1998).

[65] K. Soetaert, T. Petzoldt, F. Meysman, Marelac: A tool for aquatic sciences (R package) 2016; https://cran.r-project.org/web/packages/marelac/marelac.pdf.

[66] K. Soetaert, A. F. Hofmann, J. J. Middelburg, F. J. R. Meysman, J. Greenwood, The effect of biogeochemical processes On pH. Mar. Chem. 106( 1–2 SPEC. ISS.), 380–401 (2007).

[67] E. S. Egleston, C. L. Sabine, F. M. M. Morel, Revelle revisited: Buffer factors that quantify the response of ocean chemistry to changes in DIC and alkalinity. Glob. Biogeochem. Cycles 24, GB1002 (2010).

[68] J.-P. Gattuso, J.-M. Epitalon, H. Lavigne, J. Orr, B. Gentili, A. Hofmann, A. Proye, K. Soetaert, J. Rae, Seacarb: Seawater carbonate chemistry (2016); http://CRAN.R-project.org/package=seacarb.

